# Outcome predictors and quality of life of severe burn patients admitted to intensive care unit

**DOI:** 10.1186/1757-7241-18-24

**Published:** 2010-04-27

**Authors:** Vittorio Pavoni, Lara Gianesello, Laura Paparella, Laura Tadini Buoninsegni, Elisabetta Barboni

**Affiliations:** 1 Department of Critical Medical-Surgical Area, Section of Anesthesia and Intensive Care, Largo Palagi, 1. 50139 Firenze, Italy

## Abstract

**Background:**

Despite significant medical advances and improvement in overall mortality rate following burn injury, the treatment of patients with extensive burns remains a major challenge for intensivists. We present a study aimed to evaluate the short- and the long-term outcomes of severe burn patients (total body surface area, TBSA > 40%) treated in a polyvalent intensive care unit (ICU) and to assess the quality of life of survivors, one year after the injury using the EuroQol-5D (EQ-5D) questionnaire.

**Methods:**

A prospective-observational study was performed in an ICU of a University-affiliated hospital. Logistic regression analysis was used to identify the factors predicting in-hospital mortality. The EQ-5D questionnaire was used to asses participant's long term self-reported general health.

**Results:**

During a period of five years, 50 patients participated in the study. Their mean age was 53.8 ± 19.8; they had a mean of %TBSA burned of 54.5 ± 18.1. 44% and 10% of patients died in the ICU and in the ward after ICU discharge, respectively. Baux index, SAPS II and SOFA on admission to the ICU, infectious and respiratory complications, and time of first burn wound excision were found to have a significant predictive value for hospital mortality. The level of health of all survivors was worse than before the injury. Problems in the five dimensions studied were present as follows: mobility (moderate 68.5%; extreme 0%), self-care (moderate 21%; extreme 36.9%), usual activities (moderate 68.5%; extreme 21%), pain/discomfort (moderate 68.5%; extreme 10.5%), anxiety/depression (moderate 36.9%; extreme 42.1%).

**Conclusions:**

In severe burn patients, Baux index, severity of illness on admission to the ICU, complications, and time of first burn wound excision were the major contributors to hospital mortality. Quality of life was influenced by consequences of injury both in psychological and physical health.

## Background

The treatment of patients with extensive burns remains a major challenge, even with advances in burn care over recent decades [1]. Some publications [2,3] have suggested that survival rates reach 50% in young adults sustaining a Total Body Surface Area (TBSA) burned of 80% without inhalation injury. Recent U.S. data indicate a 69% mortality rate among patients with burns over 70% of TBSA [4].

Burn patients are an heterogeneous population, with wide variation in age, mechanism of injury, depth and site of burn and a different co-morbidity [5]. Attempts to provide valid and objective estimates of the risk of death following burn have a long and extensive history, yet little has changed during the time [2].

Hence it is important to identify injury- and treatment-related factors influencing survival of patients with severe burns.

A number of factors outside the control of the burn service may also influence outcome, including motivation of the patient, pre-burn psychological morbidity, family support and socio-economic background [6]. Burn injury may affect all aspects of human life, leaving survivors with a variety of physical and psychosocial handicaps. In addition, altered appearance and stigmatization may represent a threat to patient social life [7]. Burn survivors often have a challenging and protracted recovery process. Somatic symptoms are generally persistent and psychiatric disorders such as post-traumatic stress disorders (PTSD) and depression are relatively frequent [8]. To better understand the impact of morbidity and consequences of thermal injury and to evaluate clinical programs for treatment and follow-up, assessment of burn patient health status and quality of life have been advocated [9,10]. One of the few specific instruments that were used to support such an effort was the Burn Specific Health Scale (BSHS), validated and finalised into an abbreviated 80-item version. This questionnaire was designed to assess the post-injury adjustment by means of health-related quality of life in adult burn survivors. It includes both physical and psychosocial domains. Nevertheless this questionnaire is rather long and some authors have criticized it as being laborious to use [11]. The instrument must aim to be simple and easy to use. One such instrument could be the EuroQol-5D (EQ-5D) [12] which is a simple questionnaire used by a number of patients with specific diseases, including critically ill patients [13]; it is validated in burn patients [14] and used to provide information on the costs of the different type of burn treatment [15].

The primary aim of this study was prospectively to evaluate the short and the long term mortality of severe burn patients (TBSA > 40%) [16] admitted to the ICU and requiring ventilatory support; we also identified which clinical factors at the time of injury would predict in-hospital mortality. The second objective was to determine their health related quality of life (HR-QoL) one year after the injury, using the EQ-5D questionnaire.

## Methods

This study was performed in the Department of Intensive Care (ICU) of academic hospital of Padova. In this hospital, that represents the reference center for adult burn patients throughout the north east of Italy, there is a specialized burn unit for non intubated burn patients attended by staff plastic surgeons with burns care experience and a polyvalent ICU (16 beds) with two isolated-single bed rooms dedicated to ventilated severe burn patients under the supervision of intensivists. The ICU has four medical staff members participating in continuing medical education of burn patients, mainly nurses (two nurses for one patient) and nursing auxiliary staff members (one for each patient).

After obtaining the approval of the Research Ethical Committee of University-Hospital of Padova and the written consent of the patients or their relatives, during a 5-year period (from 1 January 1999 to 31 December 2003), all adult severe burn patients (TBSA > 40%) admitted to the ICU and requiring mechanical ventilation (MV) were prospectively included in the study. Demographic data (age, gender), severity of illness (SAPS II, Simplified Acute Physiology Score and SOFA, Sequential Organ Failure Assessment) on admission, medical comorbidities using Charlson Comorbidity Index Score [17], % TBSA burned, Baux index (age plus %TBSA burned), degree of burn, location of burns, aetiology of injury, presence of inhalation injury, timing of wound excision and grafting, length of ICU stay, short term mortality (ICU and hospital mortality), were recorded for each patient. Inhalation injury was defined by the following: history of burn occurring in an enclosed space; singeing of facial hair; soot in the oral pharynx; inflammation of the lower airway on fiberoptic broncoscopy [18,19]. Timing of wound excision and grafting was decided by surgeons and intensivists based on evaluation of burns and patient's resuscitation.

The records of interest for this study included infectious and non-infectious complications. Non infectious complications were categorized based on organ system as follows: cardiovascular (cardiogenic shock, heart failure, dysrhytmia requiring pharmacological treatment), pulmonary (pulmonary embolism, Acute Respiratory Distress Syndrome, Chronic Obstructive Pulmonary Disease, pneumothorax), neurologic (anoxic brain injury, seizure), hematologic (deep venous thrombosis, heparin-induced thrombocytopenia, gastrointestinal bleeding), and renal (acute renal failure requiring dialysis or haemofiltration). Infectious complications included sepsis, septic shock, bloodstream infections, catheter-based infections, urinary tract infections and pneumonia.

### Follow-up and health related quality of life measurement

All patients discharged from the hospital and their family were asked to report any long-term complications, such as mortality.

One year after discharge, a telephone interview was carried out with survivors to discover their quality of life. Patients, who refused or were unable to complete the questionnaire, were excluded from the study. The HR-QoL was assessed using the descriptive EQ-5D questionnaire that was administered by the same author.

The EQ-5D questionnaire was developed in 1990 and further modified to the current version with five dimensions in 1991 by the EuroQol Group [12,20]. It comprises two parts: the EQ-5D self-classifier, a self-reported description of health problems according to a five dimensional classification (i.e. mobility, self-care, activities, pain/discomfort and anxiety/depression), and the EQ VAS, a self-rated health status using visual analogue scale (VAS), similar to a thermometer, that records the perceptions of a participant's current overall health. The scale is from 0 (the worst imaginable state of health) to 100 (the best imaginable state). In both, the time frame is the day of responding. The "perceived current health status" was evaluated with the question: "Compared with my general level of health before the burn injury, your health state today is better/the same/worse".

### Statistical analysis

All analysis was performed with the statistical package SPSS for Windows (version 11,0; SPSS, Chicago, II). Results were presented as the mean ± standard deviation (SD) (continuous variables) or percentage (categorical variables). T-test (for continuous variables) and chi-square test or Fisher's exact test when expected frequencies were too small (for categorical variables) were used to compare the clinical characteristics of the ICU survivors and ICU non-survivors. Statistical significance was considered if p < 0.05.

Multivariate analysis was performed to evaluate the factors influencing in-hospital mortality.

The cumulative survival rate of the patients was plotted as a Kaplan-Meier analysis. For comparison, the cumulative survival rate of the normal population was calculated with an age- and gender-matched population using death probability tables published by ISTAT (Istituto Nazionale di Statistica) [21].

## Results

During a period of five years, 50 patients (28 males and 22 females) were admitted to the ICU after severe burn injury. Any patient refused to participate in the study. The mean age of patients was 53.8 ± 19.8. The average percentage of the TBSA burned was 54.5 ± 18.1 and the Baux score was 108.4 ± 21.3. Most of the patients (88%) had suffered third degree burns. The SAPS II and SOFA on admission were respectively 32.2 ± 13.8 and 3.9 ± 3.8. The mean of Charlson comorbidity score was 1.1 ± 1.1.

Cause of injury was the fire in 46 patients (92%) and chemicals in 4 patients (8%). Most of the patients (22 patients) (44%) had burns to the head associated to the upper and lower extremities; burn to the head, face and neck were present in 8 (16%) patients.

Infectious complications were overall the most common complications, occurring in 27 (55%) of all patients. The most common non infectious complications were respiratory failure in 24 patients (48%): acute respiratory distress syndrome (16 patients), Chronic Obstructive Pulmonary Disease (4 patients), pulmonary embolism (2 patients) and pneumothorax (2 patients). Cardiovascular complications occurred in 16 patients (32%): dysrythmia in 8 patients, heart failure in 4 patients, cardiovascular shock in 4 patients. Renal, hematologic, and neurological complications occurred in 30%, 2% and 2%, respectively of the overall population.

The average length of ICU stay was 23 ± 26.4 days. The patients were intubated and underwent MV because of inhalation injury (21 patients) or upper airways edema (29 patients). Twenty-two patients (44%) died in the ICU, most of them for infectious complications. ICU non-survivor patients died at a mean of 30.9 ± 33.6 days in the ICU. The ICU survivors had significantly lower SAPS II, SOFA on admission, %TBSA burned, Baux index, presence of third degree burns, inhalation injury, infectious and respiratory complications, length of MV, time of first burn wound excision and length of ICU stay than ICU non-survivor patients (Table 1).

**Table 1:** Clinical characteristics of ICU burn patients

	Total (n = 50)	Survivors (n = 28)	Non-survivors (n = 22)
Sex (M/F)	28/22	16/12	15/7
Age (years)	53.8 ± 19.8	50.1 ± 19.4	58 ± 20
SAPS II	32.2 ± 13.8	25.9 ± 11.1	40 ± 13.2*
SOFA	3.9 ± 3.8	2.3 ± 1.8	6 ± 4.6*
Charlson comorbidity index score	1.1 ± 1.1	0.9 ± 1.2	1.2 ± 1.2
TBSA (%)	54.5 ± 18.1	47.6 ± 12.4	63.0 ± 20.8*
Third degree (%)	44 (88)	22 (78.5)	22 (100)*
Baux index	108.4 ± 21.3	97.8 ± 13.7	121.4 ± 13.8*
Burn site: head, face and neck (%)	8 (16)	4 (14)	4 (18)
head+upper/lower extrem (%)	22 (44)	15 (54)	7 (32)
thorax, abdomen (%)	13 (26)	9 (32)	4 (18)
head+upp/low.ext+thor+abd.(%)	7 (14)	0	7 (32)*
Aetiology of injury: flame (%)	46 (92)	24 (85.7)	22 (100)
chemical(%)	4 (8)	4 (14.3)	0*
Inhalation injury (%)	21 (42)	9 (32.1)	12 (54.5)*
Complications: infectious	27 (55%)	6 (21%)	21(95%)*
respiratory	24 (48%)	8 (28%)	16 (72%)*
renal	15 (30%)	8 (28%)	7 (32%)
cardiovascular	16 (32%)	8 (28%)	8 (37%)
haematologic	1 (2%)	0	1 (4%)
neurologic	1 (2%)	0	1 (4%)
Length of MV (days)	8.7 ± 3.2	15.3 ± 21.7	37.6 ± 37.5*
Time of first escharectomy (days)	13.1 ± 7.6	10.3 ± 6.0	17.2 ± 7.4*
Length of ICU stay (days)	23 ± 26.4	15.6 ± 14.9	30.9 ± 33.6*
Length of hospital stay (days)	36.1 ± 27.1	41.6 ± 18.9	30.9 ± 33.6

Values are means ± SD or number of patients and percentages

* p value < 0.05

Five patients (10%) died in the ward after ICU discharge. One of these died a cause of pulmonary edema and he was transferred to the Department of Cardiology. An other one died of heart failure and sudden cardiac arrest. Three patients died a cause of wound infections and they were treated on department of plastic surgery under surgical direction, next to the ICU.

Baux index, SAPS II and SOFA on admission to the ICU, infectious and respiratory complications, and time of first burn wound excision were significant predictors of the hospital mortality (Table 2). However, when considering patients with TBSA burned ≥50%, time of first escharectomy (OR 2.33, 95% CI: 1.25-4.33, p = 0.01) and infections (OR 10.54, 95% CI: 1.85-54.80, p = 0.008) seems to be the most important risk factors influencing hospital mortality.

**Table 2:** Multivariate analysis for factors influencing in-hospital mortality.

Variable	Odd ratio	95% CI	p Value
Age	1.038	(0.99-1.08)	0.08
SAPS II	1.209	(1.05-1.38)	0.006
SOFA	1.597	(1.04-2.44)	0.031
Charlson score	1.283	(0.67-2.45)	0.45
TBSA (%)	1.162	(0.86-1.55)	0.31
TBSA ≥ 50%	19.50	(4.35-41.75)	<0.0001
Degree of burn	0.361	(0.04-3.96)	0.40
Baux index	1.071	(1.01-1.13)	0.01
Infectious complications	10	(1.79-55.62)	0.008
Respiratory complications	6.75	(1.31-34.56)	0.021
Renal complications	5.454	(0.54-54.27)	0.479
Time of first escharectomy	1.928	(1.10-3.37)	0.021

**Figure 1:**
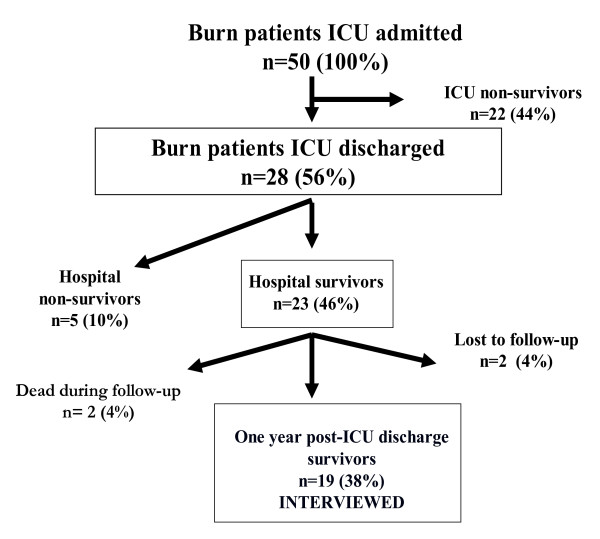
**Outcome of 50 patients with severe burn after admission ICU between January 1,1999 and December 31, 2003; follow-up process was in December 2004**.

Of the 23 patients who were discharged from hospital, two were unreachable and two died during the follow-up period from pulmonary infection and acute myocardial infarction. Figure 1 shows the follow-up process.

**Figure 2:**
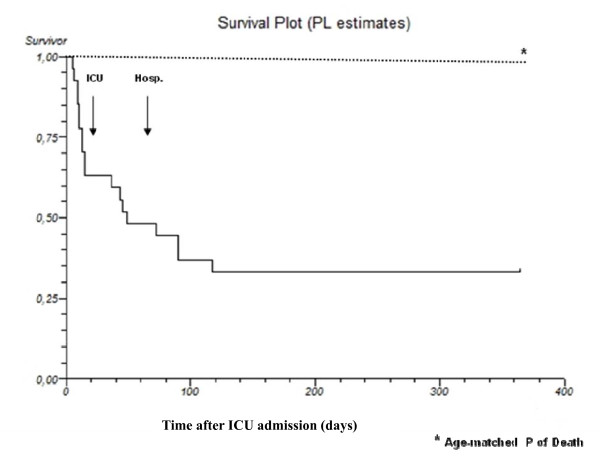
**Cumulative survival rate from ICU admission to one year after ICU discharge as plotted by Kaplan Meier compared to normal population**.

After hospital discharge, during one year follow-up period, the observed median mortality in burn patients admitted to the ICU did not increase as compared with the expected mortality of the age- and gender-matched general Italian population (4% vs 2%, p = NS) (Figure 2). None of considered parameters was associated with increased mortality risk within 1 year following discharge.

Nineteen patients were interviewed (11 males and 8 females). Table 3 shows the clinical characteristics of interviewed patients. The EQ VAS was 50 (minimum 10, maximum 80). At the time of interview the level of health of all patients was worse than previously to the injury.

**Table 3:** Characteristics of interviewed patients

Sex (M/F)	11/8
Age (years)	46.3 ± 14.9
SAPS II	23.3 ± 8.7
SOFA	2.5 ± 1.5
Charlson comorbidity index score	0.8 ± 1.1
TBSA (%)	50.1 ± 15.5
Third degree (%)	17 (89.4%)
Baux index	96.4 ± 20.8
Burn site:	
head, face and neck (%)	3 (15.7)
head+upper/lower extremities (%)	10 (52.7)
thorax, abdomen (%)	6 (31.6)
Aetiology of injury:	
flame (%)	19 (100)
chemical (%)	0
Inhalation (%)	5 (26.3)
Length of MV (days)	8.7 ± 3.2
Time of first escarectomy (days)	9.6 ± 5.2
Length of ICU stay (days)	11.7 ± 8.1
Length of hospital stay (days)	40 ± 12.5

Values are means ± SD or number of patients (n) and percentages

Ten patients (52.6%) reported an extreme problem in at least one dimension. The most frequently reported extreme symptom was anxiety/depression 8/19 = 42.1%.

Thirteen patients had moderate problems with mobility and, in contrast, no-one reported extreme problems with mobility. These percentages increased when patients were asked about their self care and pain/discomfort and anxiety/depression: 57.9%, 79% and 79% respectively, reported moderate to extreme problems. Moreover, the problems most frequently reported (from moderate to extreme) were in everyday activities (89.5%).

Extreme anxiety/depression was reported by six patients with previous psychiatric problems and by two patients who were unable to use their hands after the injury; seven patients with facial deformities and burn scars on the hands suffered moderate anxiety.

**Figure 3:**
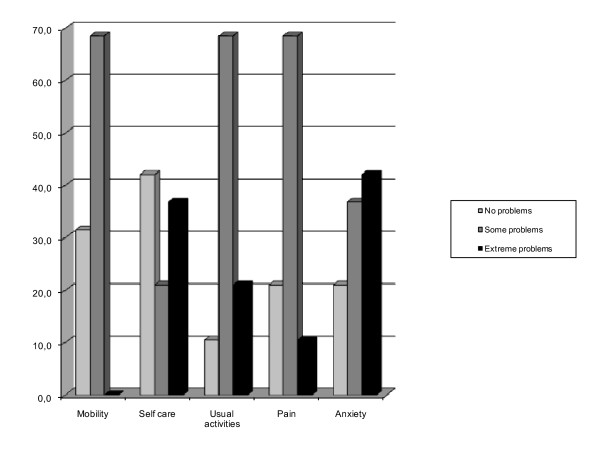
**Health related quality of life of burn patients using EuroQoL questionnaire one year after ICU discharge**. Perceived current health status: VAS score (100% scale) 50. worse (%) 100. Work: retired patients (%) 36.8. un-employed patients (%) 63.2.

In terms of main activity, none of the patients interviewed went back to work (36.8% were retired and 63.2% were un-employed) (Figure 3).

## Discussion

Even with advances in burn care over recent decades [1,22], the mortality rate remains high among severely burned patients. In our study we reported that TBSA ≥50%, presence of infections during ICU stay and "timing" of first escharectomy were indicators of hospital mortality. In particular, in the literature, early excision was associated with better outcome and shortened hospital stay [23,24]. In fact, the removal of the burn eschar potentially breaks the source of wound infection. Leaving devitalized tissue on the wound not only increased bacterial and fungal colonization, but also induced bacterial and fungal invasion into subcutaneous viable tissue [25]. Many authors have found that burn excision can be begun as soon as the initial assessment and stabilization have been completed and can be performed while resuscitation is continued [22]. Despite these findings, the literature does not answer conclusively the question of which treatment protocol is optimal. Barret and coll. [25] have demonstrated that all severe burns should be excised within 48 hours for full beneficial effects. Other studies have addressed of possible age-dependent effect on mortality. In a prospective series, Herndon et al. [26] examined burns of greater than 30 percent of TBSA. There was significantly reduced mortality with early excision (within 72 hours) for patients 17 to 30 years of age who had not sustained inhalation injury. No difference in mortality could be demonstrated for patients older than 30 years. Similarly, Kirn and coll. [27] concluded that elderly burn patients (70 years or older) did not benefit from early (minor than 7 days) eschar excision and grafting. In the present study, patients who underwent early wound excision (within 10 days) had better prognosis.

Our population was composed by severe burn patients (TBSA > 40%) with a mean age of 54 years and with low number of comorbidities. The mortality rate was higher (54%) than other studies.

Wang and coll. [22], identified an overall mortality rate of 30.4% among 102 cases of severely burned patients reviewed. The patient cohort was younger than our population (36.7 ± 11.9 vs 53.8 ± 19.8). Akerlund and coll. [28], in a large national-wide epidemiological study of burned patients, reported a low mortality rate (3%). Unfortunately, the data on burn size and depth were not found and usable as a large number of patients in this database lacked such information.

In our population infectious complications were overall the most common complication, occurring in 55% of all patients. Perhaps, the late surgical wound excision may have increased the death rate due to high incidence of wound sepsis and pneumonia. Moreover, outcomes of burn care are essentially multifaceted and complex. A specialized burn clinic could be a better predictor of good results than a polyvalent ICU because it coordinates adequate therapy with isolation of patients and reconstructive surgery. According to this analysis of data available in the National Burn Repository [29], burn mortality depends not only on patient characteristics but also where the patient is treated.

An ICU specialized on treatment of severe burn patients, even with respiratory failure, could improved outcome, but the cost-effectiveness should be evaluated.

If we consider the long term mortality, only two patients died during the follow-up period for reasons apparently not related to thermal injury. Moreover, in the patients who survived to injury and were discharged alive from the hospital, the risk of one- year mortality was not significantly different from that of the normal population. Lundgren et al. [30] recently reported correlation between baseline medical comorbidities along with age ≥75 and 1-year mortality. Lionelli et al. [31] observed that the risk of mortality was increased by a factor of 1.1 for each additional year of age, independent of the presence of an additional inhalation injury or TBSA. When age and inhalation injury were held constant, and burns were stratified by TBSA, a statistically significant increase in mortality was seen as TBSA surpassed 20%. Furthermore, when comparing mortality rates for burn patients with TBSA between 21% and 30% versus patients with 11% and 20% TBSA, mortality rates were two to three times higher.

In addition to mortality, we examined the health-related quality of life. Recently, it has been shown that perceived health problems after burn injury can persist for several decades [32]. Burn injuries were associated with long-term health problems with a variety of complications including physical limitation, psychological and social disturbance [8]. According to other studies [33], that have used the BSHS, the evaluation of post-trauma quality of life revealed significant impairment of patients' functional abilities such as in mobility and in everyday activities.

We found that, in severe burn patients, the QoL was influenced by consequences of injury both in psychological and physical health; one year after the injury most had some difficulties carrying out everyday activities and suffered pain and anxiety. Shakespeare [34], in a population with burn injury less than 20% of body surface area, at three months after discharge from treatment, reported from little to a lot of pain in 47% of the patients. In our population, one year after the injury, from moderate to severe pain was present in the majority of responders (79% of the patients). In particular, if asked, mobility impairment as consequence of pain seems to be the most important factor. This prolonged problem of psychological domains was unexpected and it has to be considered.

Between 13% and 23% of patients develop depression, and 13-45% develop post-traumatic stress disorder (PTSD) after hospital discharge [35]. It has been suggested that there is a correlation between the site of burn injury and the psychological impairment [16]. A high percentage of our patient population (47.3%) presented burn scars to the hands and facial deformities. Moreover, 31.5% of the patients with anxiety, suffered from previous psychiatric problems. That could explain the high incidence of psychological disturbances in our burn population.

Unlike Anzarut and coll. [10], that showed how survivors of burn injury reported a good quality of life, our results suggest that one year after the injury the self perceived health status remains worse than status pre-injury. Other studies [36-38] identified a correlation between the return to work and HR-QoL, trauma-related physical and psychological health. Our results identifying the persistency of worse HR-QoL, could be explain by the fact that, one year after injury, the most of them were still un-employed.

The study's limitation was the small size of the studied population because this type of burn patient is rare. We have considered a particular burn population with extensive burn injury requiring admission to ICU a cause of severity of trauma. Our findings identify specific areas requiring further investigation, perhaps through multicenter studies because of high cost in therapeutic actions and low survival rate of considered population.

## Conclusions

Burn care requires multiple disciplines working together as a cohesive team to ensure optimal outcomes. This should prompt a discussion on the treatment and on organisation to explain the differences in overall mortalities between the studies. Finally, the HR-QoL one year after the accident is low and it is influenced by consequences of injury both in psychological and physical health. Severe burns remained a burden for the society because none of them returned to work after one year. Those findings are hard to say, but the knowledge of them could help clinicians in informing patients and caregivers.

On the basis of these data, we suggest that burn system look beyond the acute hospital phase and make efforts to provide care and psychological support to burn patients during and after hospital discharge for improve the outcome both in terms of mortality and quality of life.

## Competing interests

The authors declare that they have no competing interests.

## Authors' contributions

VP performed data analysis and interpretation and revised the manuscript critically for important intellectual content. LG drafting the manuscript and participated in data analysis and interpretation. LP interpreted data and made contributions to conception and design of the study. LTB participated to conception and design of the study. EB has made substantial contributions to acquisition of data. All authors read and approved the manuscript.
